# Reproductive and hormonal factors and risk of renal cell carcinoma among women in the European Prospective Investigation into Cancer and Nutrition

**DOI:** 10.1002/cam4.6207

**Published:** 2023-06-03

**Authors:** Joanna L. Clasen, Rita Mabunda, Alicia K. Heath, Rudolf Kaaks, Verena Katzke, Matthias B. Schulze, Anna Birukov, Giovanna Tagliabue, Paolo Chiodini, Rosario Tumino, Lorenzo Milani, Tonje Braaten, Inger Gram, Marko Lukic, Leila Luján‐Barroso, Miguel Rodriguez‐Barranco, María‐Dolores Chirlaque, Eva Ardanaz, Pilar Amiano, Jonas Manjer, Linnea Huss, Börje Ljungberg, Ruth Travis, Karl Smith‐Byrne, Marc Gunter, Matthias Johansson, Sabina Rinaldi, Elisabete Weiderpass, Elio Riboli, Amanda J. Cross, David C. Muller

**Affiliations:** ^1^ Department of Epidemiology and Biostatistics School of Public Health, Imperial College London London UK; ^2^ Division of Cancer Epidemiology German Cancer research Center (DKFZ) Heidelberg Germany; ^3^ German Institute of Human Nutrition Potsdam‐Rehbruecke Nuthetal Germany; ^4^ Institute of Nutritional Science, University of Potsdam Nuthetal Germany; ^5^ German Center for Diabetes Research (DZD) Muenchen‐Neuherberg Germany; ^6^ Department of Nutrition, Harvard T.H. Chan School of Public Health Boston Massachusetts United States; ^7^ Cancer Registry Unit, Department of Research Milan Italy; ^8^ Medical Statistics Unit University L. Vanvitelli Naples Italy; ^9^ Hyblean Association for Epidemiological Research (AIRE ‐ONLUS) Ragusa Italy; ^10^ Department of Clinical and Biological Sciences University of Turin Turin Italy; ^11^ Department of Community Medicine UiT The Arctic University of Norway; ^12^ Faculty of Health Sciences, Department of Community Medicine University of Tromsø, The Arctic University of Norway Tromsø Norway; ^13^ Catalan Institute of Oncology (ICO‐IDIBELL), Cancer Epidemiology Research Program, Unit of Nutrition and Cancer L'Hospitalet de Llobregat Spain; ^14^ Escuela Andaluza de Salud Pública (EASP) Granada Spain; ^15^ Instituto de Investigación Biosanitaria ibs.GRANADA Granada Spain; ^16^ Centro de Investigación Biomédica en Red de Epidemiología y Salud Pública (CIBERESP) Madrid Spain; ^17^ Department of Epidemiology, Regional Health Council, IMIB‐Arrixaca Murcia University Murcia Spain; ^18^ Navarra Public Health Institute Pamplona Spain; ^19^ IdiSNA, Navarra Institute for Health Research Pamplona Spain; ^20^ Ministry of Health of the Basque Government, Sub Directorate for Public Health and Addictions of Gipuzkoa San Sebastian Spain; ^21^ Biodonostia Health Research Institute Epidemiology of Chronic and Communicable Diseases Group San Sebastián Spain; ^22^ Department of Surgery, Skåne University Hospital Malmö Lund University Malmö Sweden; ^23^ Department of Clinical Sciences Malmö Lund University Malmö Sweden; ^24^ Department of Surgery Helsingborg Hospital Helsingborg Sweden; ^25^ Department of Surgical and perioperative sciences, Urology and Andrology Umeå University Sweden; ^26^ Cancer Epidemiology Unit, Nuffield Department of Population Health University of Oxford Oxford UK; ^27^ International Agency for Research on Cancer Lyon France; ^28^ Department of Epidemiology and Biostatistics, School of Public Health MRC‐PHE Centre for Environment and Health, Imperial College London London UK

## Abstract

**Background:**

Renal cell carcinoma (RCC) is twice as common among men compared with women, and hormonal factors have been suggested to partially explain this difference. There is currently little evidence on the roles of reproductive and hormonal risk factors in RCC aetiology.

**Materials & Methods:**

We investigated associations of age at menarche and age at menopause, pregnancy‐related factors, hysterectomy and ovariectomy and exogenous hormone use with RCC risk among 298,042 women in the European Prospective Investigation into Cancer and Nutrition (EPIC) study.

**Results:**

During 15 years of follow‐up, 438 RCC cases were identified. Parous women had higher rates of RCC compared with nulliparous women (HR = 1.71, 95% CI 1.18, 2.46), and women who were older at age of first pregnancy had lower rates of RCC (30 years + vs. <20 years HR = 0.53, 95% CI 0.34, 0.82). Additionally, we identified a positive association for hysterectomy (HR = 1.43 95% CI 1.09, 1.86) and bilateral ovariectomy (HR = 1.67, 95% CI 1.13, 2.47), but not unilateral ovariectomy (HR = 0.99, 95% CI 0.61, 1.62) with RCC risk. No clear associations were found for age at menarche, age at menopause or exogenous hormone use.

**Conclusion:**

Our results suggest that parity and reproductive organ surgeries may play a role in RCC aetiology.

## Graphical abstract

Reproductive and hormonal factors are known to be associated with the risk of several cancers in women, but their role in kidney cancer is unclear. Despite the higher incidence of kidney cancer in men, its aetiology in women deserves further exploration. Using data from a large, prospective cohort study, we have found evidence of associations between pregnancy‐related exposures as well as hysterectomy and bilateral ovariectomy with risk of kidney cancer.

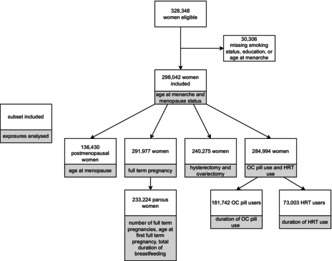

## 1 INTRODUCTION

Kidney cancer burden has increased over recent decades, and while incidence is highest among men, its impact is not negligible among women, being the 10th most common cancer in women in Europe.^1^ Most kidney cancers are renal cell carcinomas (RCC), accounting for 85%–90% of diagnoses, and of these, around 70% are clear cell renal cell carcinoma (ccRCC).^2^, ^3^ While RCC is twice as common among men compared with women, there is variation across histological subtypes. In comparison to ccRCC, papillary RCC cases are less likely to be female, while chromophobe RCC cases are more likely to be female.^3^ In addition to sex, established risk factors for RCC include older age, cigarette smoking, obesity and hypertension.^2^

Given the sex difference in kidney cancer, it has been suggested that hormonal factors might play a role. Women are exposed to fluctuating patterns of sex hormone concentrations (particularly oestrogens) during their lifetime, largely dependent on the timing of menstruation, pregnancy and menopause. There are several mechanisms through which oestrogen could plausibly be involved in the prevention or promotion of tumour development, including the regulation of apoptosis and genes involved in cell proliferation.^4^ Therefore, pregnancy and other factors related to oestrogen and progesterone exposure, including exogenous hormone use and surgical removal of reproductive organs, are commonly proposed potential factors involved in cancer development in women and might be relevant for cancers with a sex difference in incidence rates.

The roles of these exposures have been extensively studied in some cancers including breast and ovarian cancer, for which nulliparity is an established risk factor.^5^ However, preliminary evidence suggests that kidney cancer does not follow the same trend, and higher parity may be associated with greater RCC incidence.^5^ Kidney‐specific physiological changes in pregnancy need to be considered to assess the role of reproductive factors in RCC risk. During pregnancy, kidneys increase in size due to fluid retention, and the glomerular filtration rate increases by 50%. These changes are likely caused by a combination of hormonal and mechanical factors.^6^ Aside from pregnancy, oestrogen‐related pathways have been implicated in RCC aetiology because there is a strong positive association between obesity and RCC risk, and adipose tissue is a source of oestrogen.^7^

The role of reproductive and hormonal risk factors in RCC aetiology remains unclear, and evidence on variability across histological subtypes is sparse and inconclusive. We aimed to examine the associations of age at menarche and age at menopause, pregnancy‐related factors, hysterectomy and ovariectomy and exogenous hormone use with RCC risk (overall and ccRCC) among women in the European Prospective Investigation into Cancer and Nutrition (EPIC) cohort.

## 2 METHODS

### 2.1 Study population

EPIC is an ongoing multi‐centre cohort study designed to investigate the relationships between dietary, metabolic, genetic and lifestyle factors and the incidence of cancer and other chronic diseases in adults from 23 centres in 10 European countries (Denmark, France, Germany, Greece, Italy, the Netherlands, Spain, Sweden, Norway and the United Kingdom).^8^ The study protocol has been described in detail previously.^8^, ^9^ Briefly, most participants were recruited from the general population, with some centres recruiting blood donors or members of health insurance programmes. Approximately 520,000 participants including around 370,000 women, mostly aged between 35 and 70 years, were enrolled between 1992 and 2000. All participants provided written informed consent. The Institutional Review Board of the International Agency for Research on Cancer and the local ethics committees approved the study.

### 2.2 Ascertainment of outcome

Incident cases of RCC were identified via population‐based cancer registries or through active follow‐up (via linkage with medical and health insurance records or contacting participants or their next of kin).

Women diagnosed with an incident histologically confirmed RCC (Second revision of the International Classification of Diseases for Oncology [ICD‐O‐2] code C64.9) were identified as cases. Morphology code 8310/3 was used to identify ccRCC cases.

### 2.3 Exposure assessment

Hormonal and reproductive history, including age at first and last menstrual cycle, menopause status, number of full‐term pregnancies, age at first pregnancy, breastfeeding duration, hysterectomy and ovariectomy, use of oral contraceptive pills (OCs) and use of hormone replacement therapy (HRT) were assessed using standardised questionnaires at the baseline visit. Reproductive history information was collected on all women except for a subset of participants in The Netherlands and Sweden. History of hysterectomy and ovariectomy was collected at most centres but is incomplete for participants recruited in Sweden and Norway (history of hysterectomy missing for 87% of women in Sweden and 25% in Norway; history of ovariectomy missing for 98% of women in Sweden and 99% in Norway). Menopausal status was defined based on menstrual status in the past 12 months, use of exogenous hormones and age, as detailed previously.^10^

The questionnaires additionally collected information on smoking habits, educational attainment and medical history, including hypertension. Height and weight were self‐reported or measured by trained staff following uniform procedures, depending on the centre.

### 2.4 Statistical methods

Cox proportional hazards models were used to estimate hazard ratios (HRs) and 95% confidence intervals (CIs) for incident RCC in relation to age at menarche (<12, 12, 13, 14, 15+ years), menopause status (premenopausal, perimenopausal, postmenopausal), age at menopause (<46, 46–48, 49–51, 52–54, 55+ years), any full‐term pregnancy (no, yes), number of full‐term pregnancies (among parous women; 1, 2, 3, 4+), age at first full‐term pregnancy (among parous women; <20, 20–24, 25–29, 30+ years), total duration of breastfeeding (among parous women; 0, <3, 3–8, 9+ months), hysterectomy (no, yes), ovariectomy (no, bilateral, unilateral), ever use of OC pill (no, yes), HRT use (never, past, current), duration of OC pill use (among users; <2, 2–5, 6–10, 11+ years) and duration of HRT use (among ever users; <2, 2–5, 6+ years). Age was used as the underlying timescale for all Cox models. In constructing the models, time of entry into the study was age at recruitment, and time of exit was the age at RCC diagnosis, death, loss to follow‐up or censoring, whichever occurred first. The end of follow‐up was defined as the latest date of complete follow‐up (for cancer diagnosis and vital status), which varied across centres and was no later than November 2015. Schoenfeld residuals were used to investigate the proportional hazards assumption, and there was no evidence of violation. The first set of models were stratified by country and adjusted for body mass index (BMI, continuous), smoking status (never, former, current) and highest level of education (none or primary school, secondary school, technical/professional school, longer education). A second set of models were additionally adjusted for age at menarche and menopause status to investigate the role of exposures after accounting for these key markers of menstrual cycle timing, with the exception of models for surgical procedures (hysterectomy and ovariectomy) which were additionally adjusted for age at menarche only. The final set of models were additionally mutually adjusted for the other exposure variables within three separate sets of related exposures (pregnancy, surgical procedures and exogenous hormone use). Additional models for menopause status and age at menopause were restricted to women without a reported hysterectomy or bilateral ovariectomy. All covariates were selected a priori based on a literature review. Data on participants from Greece were not available for the current analyses. In addition, participants were excluded from all analyses if they were missing smoking status, highest level of education or age at menarche. For exposures included in a mutually adjusted model, participants were excluded from all models if they had missing data for any of the variables from that set.

The analyses above were repeated for ccRCC cases, with cases of other histological subtypes and those with no subtype specified censored at the time of diagnosis.

As a sensitivity analysis, the same models (for overall RCC) were run excluding the first 3 years of follow‐up for all participants. Additionally, because some exposures of interest may be causally related to BMI at the baseline visit, we ran separate models adjusting for BMI at age 20 years (using height at baseline and self‐reported weight at age 20). Because reported weight at age 20 is not available for all participants, we also fitted models adjusted for BMI at baseline among this smaller subset of participants for comparison. Hypertension was not adjusted for in the main analyses because it may be on the causal pathway between reproductive and hormonal factors and development of RCC; however, we conducted a secondary analysis with further adjustment for hypertension status.

All analyses were performed using R version 4.1.0. Cox regression models were fitted using the survival package.

## 3 RESULTS

### 3.1 Baseline characteristics

**FIGURE 1:**
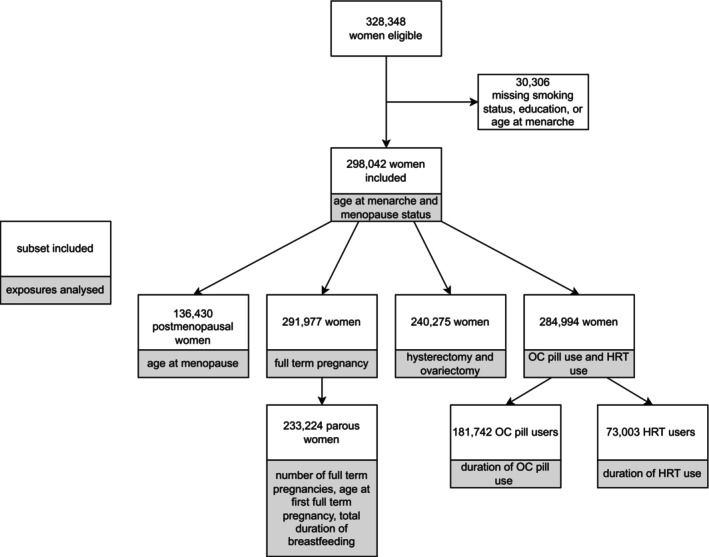
Flow chart of participants included in analyses of reproductive and hormonal risk factors for renal cell carcinoma among women in the European Prospective Investigation into Cancer and Nutrition study.

A total of 298,042 women without missing data on smoking, education or age at menarche were included in the analysis (Figure 1). During a median follow‐up of 15 years (IQR 13.5, 16.4), 438 incident RCC cases were identified. Median age at recruitment was 51 years for all participants and 56 years for cases. Almost half of participants (46%) were postmenopausal at baseline. The most common age at menarche was 13 years (26%). Most participants (83%) had at least one full‐term pregnancy, with 9% having four or more full‐term pregnancies, and the majority (78%) of first pregnancies occurring between the ages of 20–29 years. More than a third (39%) of women reported never using OC pills, and 71% of women reported no HRT use (including premenopausal women who did not answer the question on HRT use and were assumed to have never used HRT) (Table 1). Among women without missing data on hysterectomy or ovariectomy, 12% had undergone a hysterectomy and 8% either unilateral or bilateral ovariectomy; 27% of women with a hysterectomy also had a bilateral ovariectomy, and 92% of women with a bilateral ovariectomy also had a hysterectomy.

**TABLE 1:** Baseline health, lifestyle and demographic characteristics for the full cohort and incident RCC cases among women in the EPIC study.

	Total	Incident RCC cases
*N*	298,042	438
Follow‐up time (years) (median [IQR])	14.9 [13.5, 16.4]	9.5 [5.0, 12.9]
Age at recruitment (years) (median [IQR])	51.0 [45.1, 57.2]	55.8 [50.3, 61.8]
BMI (kg/m^2^) (median [IQR])	24.0 [21.8, 27.0]	25.5 [23.1, 29.0]
Smoking status (%)		
Never	166,741 (56)	221 (50)
Current	60,520 (20)	120 (27)
Former	70,781 (24)	97 (22)
Highest level of education (%)		
None or primary school completed	84,708 (28)	208 (47)
Secondary school	72,392 (24)	57 (13)
Technical/professional school	70,243 (24)	118 (27)
Longer education (incl. University deg.)	70,699 (24)	55 (13)
Country (%)		
France	61,982 (21)	4 (1)
Italy	31,082 (10)	69 (16)
Spain	25,118 (8)	42 (10)
United Kingdom	44,488 (15)	50 (11)
The Netherlands	26,965 (9)	53 (12)
Germany	27,814 (9)	54 (12)
Sweden	20,243 (7)	51 (12)
Denmark	28,173 (9)	61 (14)
Norway	32,177 (11)	54 (12)
Menopausal status (%)		
Premenopausal	103,156 (35)	63 (14)
Perimenopausal	58,456 (20)	75 (17)
Postmenopausal	136,430 (46)	300 (68)
Age at menopause (years; among postmenopausal women) (%)		
<46	22,929 (17)	66 (22)
46–48	19,632 (14)	45 (15)
49–51	31,851 (23)	59 (20)
52–54	22,481 (16)	64 (21)
55+	8600 (6)	21 (7)
Missing	30,937 (23)	45 (15)
Age at menarche (years) (%)		
<12	45,056 (15)	64 (15)
12	63,426 (21)	82 (19)
13	77,223 (26)	104 (24)
14	64,924 (22)	100 (23)
15+	47,413 (16)	88 (20)
Full‐term pregnancy (%)		
No	45,234 (15)	31 (7)
Yes	246,743 (83)	404 (92)
Missing	6065 (2)	3 (1)
Number of full‐term pregnancies (among parous women) (%)		
1	45,329 (18)	68 (17)
2	116,205 (47)	185 (46)
3	54,443 (22)	88 (22)
4+	23,098 (9)	55 (14)
Missing	7668 (3)	8 (2)
Age at first full‐term pregnancy (years; among parous women) (%)		
<20	19,738 (8)	57 (14)
20–24	106,776 (43)	150 (37)
25–29	85,776 (35)	154 (38)
30+	33,657 (14)	42 (10)
Missing	796 (0)	1 (0)
Total duration of breastfeeding (months; among parous women) (%)		
0	34,978 (14)	47 (12)
<3	49,334 (20)	77 (19)
3–8	69,503 (28)	125 (31)
9+	73,843 (30)	121 (30)
Missing	19,085 (8)	34 (8)
Hysterectomy (%)		
No	239,270 (80)	288 (66)
Yes	31,083 (10)	81 (18)
Missing	27,689 (9)	69 (16)
Ovariectomy (%)		
No	222,675 (75)	284 (65)
Bilateral	8352 (3)	28 (6)
Unilateral	9923 (3)	19 (4)
Missing	57,092 (19)	107 (24)
Ever use of OC pill (%)		
No	115,074 (39)	228 (52)
Yes	181,742 (61)	205 (47)
Missing	1226 (0)	5 (1)
Duration of OC pill use (years; among ever OC users) (%)		
<2	32,946 (18)	46 (22)
2–5	52,375 (29)	50 (24)
6–10	40,435 (22)	55 (27)
11+	39,380 (22)	42 (20)
Missing	16,606 (9)	12 (6)
HRT (%)		
Never	212,987 (71)	283 (65)
Past	21,998 (7)	46 (11)
Current	51,005 (17)	80 (18)
Missing	12,052 (4)	29 (7)
Duration of HRT use (years; among past or current HRT users) (%)		
<2	25,546 (35)	46 (37)
2–5	25,503 (35)	31 (25)
6+	16,819 (23)	37 (29)
Missing	5135 (7)	12 (10)

Abbreviations: BMI, body mass index; HRT, hormone replacement therapy; IQR, interquartile range; OC, oral contraceptive.

### 3.2 Associations of reproductive and hormonal factors with RCC risk

The data from this study did not show evidence of an association between age at menarche (HR 0.91 [95% CI 0.65, 1.27] for age 15+ compared with under 12 years) or menopausal status and RCC risk (HR 0.97 [95% CI 0.66, 1.42] for postmenopausal compared with premenopausal) (Table 2). Among postmenopausal women, age 49–51 at menopause compared with under 46 years appeared to be associated with lower RCC risk (HR 0.64 [95% CI 0.45, 0.91]), but there was no clear association for other ages at menopause, and there was less evidence of association in the subset of women without hysterectomy or bilateral ovariectomy (HR for age 49–51 vs. <46 years: 0.78 [95% CI 0.46, 1.32]).

**TABLE 2:** Hazard ratios and 95% confidence intervals for renal cell carcinoma risk by age at menarche, menopause status, and age at menopause in the EPIC study.

Exposure	Category	Number of cases	Adjusted for BMI, smoking status and education HR (95% CI)^a^	Additionally adjusted for age at menarche HR (95% CI)^b^	Restricted to women without hysterectomy or bilateral ovariectomy Number of cases HR (95% CI)^b^	
Age at menarche (years)	<12	64	Reference			
	12	82	0.90 (0.65, 1.25)			
	13	104	0.89 (0.65, 1.22)			
	14	100	0.89 (0.65, 1.23)			
	15+	88	0.91 (0.65, 1.27)			
Menopause status	Premenopausal	63	Reference	Reference	58	Reference
	Perimenopausal	75	1.04 (0.70, 1.55)	1.04 (0.70, 1.55)	49	1.14 (0.73, 1.78)
	Postmenopausal	300	0.96 (0.66, 1.41)	0.97 (0.66, 1.42)	181	0.97 (0.63, 1.48)
Among postmenopausal women						
Age at menopause (years)	<46	66	Reference	Reference	22	Reference
	46–48	45	0.81 (0.56, 1.19)	0.81 (0.55, 1.19)	29	1.06 (0.61, 1.84)
	49–51	59	0.64 (0.45, 0.92)	0.64 (0.45, 0.91)	40	0.78 (0.46, 1.32)
	52–54	64	0.97 (0.68, 1.37)	0.96 (0.68, 1.37)	44	1.13 (0.67, 1.89)
	55+	21	0.81 (0.50, 1.34)	0.81 (0.49, 1.33)	18	1.20 (0.64, 2.26)

^a^ Stratified by country and adjusted for BMI, smoking status, and education.

^b^ Stratified by country and adjusted for BMI, smoking status, education, and age at menarche.

Estimated HRs and 95% CIs of pregnancy‐related exposures with RCC risk are shown in Table 3. Having had at least one full‐term pregnancy was associated with a 1.71‐fold higher rate of RCC compared with having had no full‐term pregnancies (HR 1.71 [95% CI 1.18, 2.46], additionally adjusted for age at menarche and menopause status). We did not find evidence for an association with RCC for number of full‐term pregnancies or total duration of breastfeeding among parous women. Older age at first full‐term pregnancy was associated with lower RCC risk (HR 0.53 [95% CI 0.34, 0.82] for age 30+ compared with less than 20 years). Mutual adjustment across pregnancy‐related exposures had minimal impact on estimates.

**TABLE 3:** Hazard ratios and 95% confidence intervals for renal cell carcinoma risk by reproductive factors in the EPIC study.

Exposure	Category	Number of cases	Adjusted for BMI, smoking status and education HR (95% CI)^a^	Additionally adjusted for age at menarche and menopause status HR (95% CI)^b^	Mutually adjusted HR (95% CI)^c^
Full‐term pregnancy	No	31	Reference	Reference	
	Yes	404	1.71 (1.18, 2.47)	1.71 (1.18, 2.46)	
Among parous women only					
Number of full‐term pregnancies	1	68	Reference	Reference	Reference
	2	173	1.00 (0.75, 1.33)	1.00 (0.75, 1.33)	0.97 (0.72, 1.31)
	3	82	0.91 (0.66, 1.26)	0.91 (0.66, 1.27)	0.90 (0.63, 1.29)
	4+	54	1.12 (0.77, 1.62)	1.13 (0.78, 1.63)	1.14 (0.75, 1.72)
Age at first full‐term pregnancy (years)	<20	53	Reference	Reference	Reference
	20–24	137	0.55 (0.40, 0.76)	0.55 (0.40, 0.76)	0.56 (0.40, 0.78)
	25–29	148	0.73 (0.52, 1.02)	0.74 (0.53, 1.03)	0.73 (0.52, 1.04)
	30+	39	0.53 (0.34, 0.81)	0.53 (0.34, 0.82)	0.51 (0.32, 0.81)
Total duration of breastfeeding (months)	0	47	Reference	Reference	Reference
	<3	77	1.01 (0.70, 1.45)	1.01 (0.70, 1.45)	1.00 (0.69, 1.44)
	3–8	124	1.02 (0.73, 1.44)	1.02 (0.73, 1.44)	1.01 (0.71, 1.42)
	9+	121	0.87 (0.61, 1.23)	0.87 (0.62, 1.23)	0.84 (0.59, 1.21)

^a^ Stratified by country and adjusted for BMI, smoking status and education.

^b^ Stratified by country and adjusted for BMI, smoking status, education, age at menarche and menopause status.

^c^ Stratified by country and adjusted for BMI, smoking status, education, age at menarche, menopause status and mutually adjusted for the other pregnancy‐related exposures.

Associations of reproductive‐related surgical procedures with risk of RCC are shown in Table 4. Hysterectomy (HR 1.43 [95% CI 1.09, 1.86]) and bilateral ovariectomy (HR 1.67 [95% CI 1.13, 2.47]), but not unilateral ovariectomy (HR 0.99 [95% CI 0.61, 1.62]) were associated with higher RCC incidence compared with not having these procedures. After mutual adjustment, HRs for hysterectomy and bilateral ovariectomy remained in the direction of positive associations, but the magnitudes of both estimates were attenuated.

**TABLE 4:** Hazard ratios and 95% confidence intervals for renal cell carcinoma risk by hysterectomy and ovariectomy status in the EPIC study.

Exposure	Category	Number of cases	Adjusted for BMI, smoking status and education HR (95% CI)^a^	Additionally adjusted for age at menarche HR (95% CI)^b^	Mutually adjusted HR (95% CI)^c^
Hysterectomy	No	257	Reference	Reference	Reference
	Yes	72	1.43 (1.10, 1.87)	1.43 (1.09, 1.86)	1.32 (0.95, 1.84)
Ovariectomy	No	284	Reference	Reference	Reference
	Bilateral	28	1.68 (1.13, 2.48)	1.67 (1.13, 2.47)	1.33 (0.83, 2.14)
	Unilateral	17	1.00 (0.61, 1.63)	0.99 (0.61, 1.62)	0.88 (0.52, 1.47)

^a^ Stratified by country and adjusted for BMI, smoking status and education.

^b^ Stratified by country and adjusted for BMI, smoking status, education and age at menarche.

^c^ Stratified by country and adjusted for BMI, smoking status, education, age at menarche and mutually adjusted for ovariectomy or hysterectomy.

Associations of exogenous hormone use with RCC risk are shown in Table 5. We found no clear evidence of associations between OC pill use, HRT use or duration of OC pill or HRT use with RCC risk.

**TABLE 5:** Hazard ratios and 95% confidence intervals for renal cell carcinoma risk by exogenous hormone use in the EPIC study.

Exposure	Category	Number of cases	Adjusted for BMI, smoking status and education HR (95% CI)^a^	Additionally adjusted for age at menarche and menopause status HR (95% CI)^b^	Mutually adjusted HR (95% CI)^c^
Ever use of OC pill	No	206	Reference	Reference	Reference
	Yes	200	1.01 (0.82, 1.25)	1.01 (0.81, 1.25)	1.01 (0.81, 1.25)
HRT	Never	282	Reference	Reference	Reference
	Past	46	1.05 (0.77, 1.45)	1.06 (0.77, 1.46)	1.06 (0.77, 1.46)
	Current	78	0.93 (0.71, 1.21)	0.94 (0.71, 1.24)	0.94 (0.71, 1.24)
Among OC ever users					
Duration of OC pill use (years)	<2	46	Reference	Reference	
	2–5	50	0.82 (0.54, 1.22)	0.82 (0.55, 1.23)	
	6–10	55	1.18 (0.78, 1.76)	1.18 (0.79, 1.77)	
	11+	42	0.82 (0.52, 1.28)	0.82 (0.52, 1.27)	
Among HRT past or current users					
Duration of HRT use (years)	<2	46	Reference	Reference	
	2–5	31	0.59 (0.37, 0.94)	0.63 (0.39, 1.01)	
	6+	37	0.94 (0.59, 1.50)	1.00 (0.62, 1.62)	

^a^ Stratified by country and adjusted for BMI, smoking status and education.

^b^ Stratified by country and adjusted for BMI, smoking status, education, age at menarche and menopause status.

^c^ Stratified by country and adjusted for BMI, smoking status, education, age at menarche, menopause status and mutually adjusted for HRT or OC use.

Analyses of ccRCC included 156 cases (Table S1). Because of the small number of cases, most risk estimates for ccRCC were accompanied by high uncertainty (Supplementary Tables S2–S5). In contrast to overall RCC, we found evidence of lower ccRCC risk for postmenopausal compared with premenopausal women (HR 0.52 [95% CI 0.29, 0.93]) (Table S2), but no associations for hysterectomy (HR 1.02 [95% CI 0.60, 1.73]) or ovariectomy (bilateral: HR 0.88 [95% CI 0.36, 2.18]) with ccRCC risk (Table S4).

Results were similar after excluding the first 3 years of follow‐up (Tables S6–S9). There were no notable differences in results from models adjusted for BMI at age 20 years compared to BMI at baseline among 137,826 women who reported weight at age 20 (Table S10). Additionally, no qualitative differences were observed after further adjusting for hypertension status (Table S11).

## 4 DISCUSSION

We explored the associations between reproductive and hormonal factors and risk of RCC among women in a large, prospective European cohort including 438 cases. Overall, we found evidence to suggest that parity and reproductive organ surgeries may be associated with higher RCC risk; furthermore, older age at first pregnancy may be inversely associated with RCC. We found no strong evidence for associations between age at menarche, age at menopause or exogenous hormone use with RCC risk.

### 4.1 Summary of findings and comparison to the literature

In this study, age at menarche and menopause status were not associated with RCC risk, nor was age at menopause among postmenopausal women (after excluding participants with a hysterectomy or bilateral ovariectomy). We did find evidence of an association between menopause status and ccRCC risk (lower risk for postmenopausal compared with premenopausal women). In contrast, one previous study reported a higher risk of overall RCC for peri‐ and postmenopausal versus premenopausal women.^11^ This discrepancy may be explained by the differences in how menopausal status was defined. They included women with bilateral ovariectomy or hysterectomy in the peri‐ and postmenopausal groups, and in our study we observed that these procedures are associated with higher risk of RCC. Our results were largely consistent with previous cohort studies that reported no association between age at menarche^11^, ^12^, ^13^, ^14^ or age at menopause^12^, ^13^, ^15^ with RCC risk, however, one investigation in two cohorts of postmenopausal women found evidence of an inverse association between age at menarche and RCC risk,^15^ and one cohort study reported higher risk for younger age at menopause.^14^

We found evidence of higher RCC risk among women with at least one full‐term pregnancy compared with those with none, and an inverse association between age at first full‐term pregnancy and RCC risk. However, we found no evidence supporting a role for number of pregnancies or duration of breastfeeding in RCC risk. Our findings are consistent with a 2013 meta‐analysis that reported a relative risk of 1.23 (95% CI 1.10, 1.36) for parity versus nulliparity and kidney cancer risk.^16^ In contrast to our results, previous studies have reported higher RCC risk among women with a higher compared with lower number of births.^12^, ^13^, ^17^ Cohort studies have generally reported no association for age at first birth and RCC risk,^11^, ^15^ but several did report evidence suggestive of a possible inverse association.^13^, ^17^ In addition to the larger size and increased function of the kidneys during pregnancy, there are also several biochemical alterations, and the ureters are compressed,^6^ all of which have the potential to add physiological stress and may contribute to an increased risk of RCC development. A cohort of women in Taiwan were followed from time of first childbirth for risk of kidney cancer mortality. In contrast to the consistently null or inverse associations between age at first birth and risk of RCC diagnosis, the Taiwan study found a positive association between age at first birth and kidney cancer mortality.^18^ It is difficult to compare results between analyses of kidney cancer incidence and mortality due to the strong differences in long‐term survival by tumour stage. Cohort studies with comprehensive information on tumour stage and grade are required to investigate whether there are indeed differing associations for kidney cancer incidence and mortality, and the extent to which such differences could be due to stage at diagnosis and tumour aggressiveness.

Our findings indicated strong positive associations between hysterectomy and bilateral (but not unilateral) ovariectomy with RCC risk. These associations were attenuated after mutual adjustment, which is not surprising given the high level of overlap in participants having these surgeries (almost all women who had a bilateral ovariectomy also had a hysterectomy). Furthermore, this overlap means that caution is required in drawing conclusions on the aetiologic role of each surgery. Our results are consistent with a meta‐analysis that found a summary relative risk for kidney cancer of 1.29 (95% CI 1.16, 1.43) for women who have had a hysterectomy compared with those who have not.^19^ Subsequent studies have similarly reported a higher risk of RCC/kidney cancer associated with hysterectomy, with HRs of 1.28 (95% CI 1.03, 1.60) in the Women's Health Initiative,^20^ 1.32 (95% CI 1.11, 1.56) in a retrospective cohort study of Western Australian women,^21^ and 1.42 (95% CI 1.01, 2.00) in the Netherlands Cohort Study.^14^ In contrast to our findings, the Netherlands Cohort Study also found an association between hysterectomy and ccRCC, with an HR of 1.63 (95% CI 1.08, 2.45).^14^ Previous findings are inconclusive for ovariectomy, with several cohorts reporting no association but an estimate in the direction of higher risk of kidney cancer for those with versus without a bilateral ovariectomy,^12^, ^13^, ^20^ and one additionally stating that risk was similar for unilateral and bilateral procedures.^15^ While changes in circulating sex hormone concentrations may potentially explain the associations we found for reproductive organ surgeries, there are several other likely explanations as well. Women undergoing such surgeries may access health services more frequently and may have incidental diagnoses of asymptomatic RCC detected through imaging. Additionally, the underlying condition that led to having a hysterectomy or ovariectomy or medication use prior to or following the procedure, rather than the surgery itself, may be related to RCC development. In future studies, it would be of interest to investigate the reasons for having these surgical procedures, but detailed information of this sort is rarely collected in cohort studies.

We did not find evidence for a role of exogenous hormone use in RCC development, which is in agreement with previous findings in a European population^14^ Meta‐analyses have reported kidney cancer relative risks of 0.89 (95% CI 0.82, 0.98) and 1.08 (95% CI 0.96, 1.22) for ever users versus non‐users of OCs and HRT, respectively.^22^, ^23^ As with hysterectomy and ovariectomy, associations between RCC and exogenous hormone use may be susceptible to confounding by indication. Further insights into the relationship between hormones and RCC might be gleaned by evaluating circulating hormone concentrations in relation to RCC.

### 4.2 Strengths and limitations

A strength of this analysis was the use of data from EPIC, a large multi‐national prospective cohort with detailed information collected on a range of reproductive and hormonal risk factors as well as other relevant lifestyle and demographic characteristics. However, a limitation is that all information on reproductive and hormonal exposures was self‐reported. A validation study in the United States found that self‐reported hysterectomy history had high accuracy, but both unilateral and bilateral ovariectomy self‐reports had lower accuracy, and 19% of women reporting a unilateral ovariectomy had both ovaries removed.^24^ Age at hysterectomy or ovariectomy was not available for all applicable participants. We were therefore unable to evaluate time between these surgeries and RCC diagnosis, which is an important consideration that warrants investigation in future studies. Additionally, we only used data collected at the baseline visit and were unable to account for changes that may have occurred prior to cancer diagnoses, for example progressing from pre‐ or perimenopausal to postmenopausal status. Reverse causation is unlikely to have had a notable impact on our results because many of the exposures occurred years prior to the baseline visit for the majority of participants (age at menarche and pregnancy‐related exposures), and the results did not vary in a sensitivity analysis excluding the first 3 years of follow‐up. The only available proxy for socioeconomic status was highest level of education, and therefore, the possibility of residual confounding cannot be excluded.

Caution should be taken in generalising these results to other populations, in particular those outside of Europe and women of a younger generation who may have different exposure profiles for reproductive and hormonal factors. OC formulations have changed over time, and similarly, surgical techniques have improved and become generally less invasive.^25^, ^26^ Therefore, women who took OCs or had a hysterectomy or ovariectomy several decades ago, as is the case for many women enrolled in EPIC, may not reflect the risk carried by women who have these same exposures today.

Our investigation of histological subtypes of RCC was restricted to ccRCC only because of low case numbers for other subtypes, which is partially due to their lower incidence but also because many diagnoses in EPIC were reported as subtype ‘NOS (not otherwise specified)’. In particular, the subtype was not reported for more than half of RCC cases in the United Kingdom, Germany, Sweden and Denmark. Despite this limitation, our findings suggested possible heterogeneity in risk factors for ccRCC compared to overall RCC.

## 5 CONCLUSIONS AND FUTURE DIRECTIONS

Overall, our findings generally concur with the sparse existing evidence on risk factors for kidney cancer in women, suggesting a higher risk of RCC associated with parity, hysterectomy and bilateral ovariectomy. Our analysis was unique, as a comprehensive interrogation of related reproductive and hormonal exposures within a single study population. Additionally, we performed mutual adjustment for exposures that are likely to be highly correlated and therefore were able to address individual potential risk factors while accounting for closely related exposures. Further investigations are needed to explore the role of sex hormones and other potential physiological mechanisms involved in these relationships.

The overall findings of this investigation suggest that hormones play a less prominent role in RCC aetiology than in other cancers such as those of the breast or endometrium. In contrast to breast cancer, our results generally are consistent with a relatively weaker and inverse association between oestrogen exposure and RCC risk. This aligns with the known lower overall incidence of RCC among women compared with men, given the far higher exposure to oestrogen among women. It is possible that the role of oestrogen in RCC development may be mediated by hypertension, a known risk factor for RCC.^2^ Oestrogen is protective against cardiovascular disease, possibly due to its role in lowering blood pressure via regulation of the renin–angiotensin–aldosterone system.^27^ Nevertheless, our findings were unchanged after further adjustment for hypertension.

Due to limited data on histological subtypes, we were unable to draw strong conclusions regarding risk factors specific to ccRCC. Further studies are required to investigate the heterogeneity of risk factors across histological subtypes of RCC.

Overall, our findings provide preliminary evidence that sex hormone pathways related to reproductive and other hormonal factors may be associated with RCC risk in women and might contribute to the lower incidence of RCC among women compared with men. However, non‐hormonal causal pathways should be considered as well, for example surveillance bias and confounding by indication in the association with surgical procedures, and physiological alterations during pregnancy in the associations with pregnancy‐related exposures.

## AUTHOR CONTRIBUTIONS

**Joanna L. Clasen:** Conceptualization (equal); formal analysis (equal); investigation (equal); methodology (equal); visualization (equal); writing – original draft (equal). **Rita Mabunda:** Conceptualization (equal); investigation (equal); methodology (equal); writing – review and editing (equal). **Alicia K. Heath:** Conceptualization (equal); investigation (equal); methodology (equal); supervision (equal); writing – review and editing (equal). **Rudolf Kaaks:** Writing – review and editing (equal). **Verena Katzke:** Writing – review and editing (equal). **Matthias B Schulze:** Writing – review and editing (equal). **Anna Birukov:** Writing – review and editing (equal). **Giovanna Tagliabue:** Writing – review and editing (equal). **Paolo Chiodini:** Writing – review and editing (equal). **Rosario Tumino:** Writing – review and editing (equal). **Lorenzo Milani:** Writing – review and editing (equal). **Tonje Braaten:** Writing – review and editing (equal). **Inger Torhild Gram:** Writing – review and editing (equal). **Marko Lukic:** Writing – review and editing (equal). **Leila Lujan‐Barroso:** Writing – review and editing (equal). **Miguel Rodríguez Barranco:** Writing – review and editing (equal). **Maria Dolores Chirlaque:** Writing – review and editing (equal). **Eva Ardanaz:** Writing – review and editing (equal). **Pilar Amiano:** Writing – review and editing (equal). **Jonas Manjer:** Writing – review and editing (equal). **Linnea Huss:** Writing – review and editing (equal). **Borje Ljungberg:** Writing – review and editing (equal). **Ruth Travis:** Writing – review and editing (equal). **Karl Smith‐Byrne:** Writing – review and editing (equal). **Marc Gunter:** Writing – review and editing (equal). **Mattias Johansson:** Writing – review and editing (equal). **Sabina Rinaldi:** Writing – review and editing (equal). **Elisabete Weiderpass:** Writing – review and editing (equal). **Elio Riboli:** Writing – review and editing (equal). **Amanda J. Cross:** Writing – review and editing (equal). **David C. Muller:** Conceptualization (equal); investigation (equal); methodology (equal); supervision (equal); writing – review and editing (equal).

## FUNDING INFORMATION

This work was supported by the Imperial College London President's PhD Scholarship to Joanna L. Clasen and the Cancer Research UK Population Research Fellowship to David C. Muller. The coordination of EPIC is financially supported by International Agency for Research on Cancer (IARC) and also by the Department of Epidemiology and Biostatistics, School of Public Health, Imperial College London which has additional infrastructure support provided by the NIHR Imperial Biomedical Research Centre (BRC). The national cohorts are supported by: Danish Cancer Society (Denmark); Ligue Contre le Cancer, Institut Gustave Roussy, Mutuelle Générale de l'Education Nationale, Institut National de la Santé et de la Recherche Médicale (INSERM) (France); German Cancer Aid, German Cancer Research Center (DKFZ), German Institute of Human Nutrition Potsdam‐Rehbruecke (DIfE), Federal Ministry of Education and Research (BMBF) (Germany); Associazione Italiana per la Ricerca sul Cancro‐AIRC‐Italy, Compagnia di SanPaolo and National Research Council (Italy); Dutch Ministry of Public Health, Welfare and Sports (VWS), Netherlands Cancer Registry (NKR), LK Research Funds, Dutch Prevention Funds, Dutch ZON (Zorg Onderzoek Nederland), World Cancer Research Fund (WCRF), Statistics Netherlands (The Netherlands); Health Research Fund (FIS)—Instituto de Salud Carlos III (ISCIII), Regional Governments of Andalucía, Asturias, Basque Country, Murcia and Navarra, and the Catalan Institute of Oncology—ICO (Spain); Swedish Cancer Society, Swedish Research Council and County Councils of Skåne and Västerbotten (Sweden); Cancer Research UK (14,136 to EPIC‐Norfolk; C8221/A29017 to EPIC‐Oxford), Medical Research Council (1,000,143 to EPIC‐Norfolk; MR/M012190/1 to EPIC‐Oxford) (United Kingdom). The recruitment phase of the EPIC‐Potsdam Study was supported by the Federal Ministry of Science, Germany (01 EA 9401) and the European Union (SOC 95201408 05F02). The follow‐up of the EPIC‐Potsdam Study was supported by German Cancer Aid (70‐2488‐Ha I) and the European Community (SOC 98200769 05F02). This work was furthermore supported by a grant from the German Ministry of Education and Research (BMBF) and the State of Brandenburg (DZD grants 82DZD00302 and 82DZD03D03). AB was further supported by the German Research Foundation (DFG) individual fellowship (#BI 2427/1–1).

## CONFLICT OF INTEREST STATEMENT

The authors declare no conflict of interest.

## DISCLAIMER

Where authors are identified as personnel of the International Agency for Research on Cancer/ World Health Organization, the authors alone are responsible for the views expressed in this article and they do not necessarily represent the decisions, policy or views of the International Agency for Research on Cancer/World Health Organization.

## ETHICS APPROVAL AND CONSENT TO PARTICIPATE

EPIC was approved by the IARC Ethics Committee and by ethics committees at participating EPIC Centres. All participants provided written informed consent prior to participating. The study was performed in accordance with the Declaration of Helsinki.

## Supporting information


**Table S1:** Histological subtypes of renal cell carcinoma cases in the EPIC study
**Table S2:** Hazard ratios and 95% confidence intervals for clear cell renal cell carcinoma risk by age at menarche, menopause status, and age at menopause in the EPIC study
**Table S3:** Hazard ratios and 95% confidence intervals for clear cell renal cell carcinoma risk by reproductive factors in the EPIC study
**Table S4:** Hazard ratios and 95% confidence intervals for clear cell renal cell carcinoma risk by hysterectomy and ovariectomy status in the EPIC study
**Table S5:** Hazard ratios and 95% confidence intervals for clear cell renal cell carcinoma risk by exogenous hormone use in the EPIC study
**Table S6:** Hazard ratios and 95% confidence intervals for renal cell carcinoma risk by age at menarche, menopause status, and age at menopause in the EPIC study, excluding the first three years of follow‐up
**Table S7:** Hazard ratios and 95% confidence intervals for renal cell carcinoma risk by reproductive factors in the EPIC study, excluding the first three years of follow‐up
**Table S8:** Hazard ratios and 95% confidence intervals for renal cell carcinoma risk by hysterectomy and ovariectomy status in the EPIC study, excluding the first three years of follow‐up
**Table S9:** Hazard ratios and 95% confidence intervals for renal cell carcinoma risk by exogenous hormone use in the EPIC study, excluding the first three years of follow‐up
**Table S10:** Hazard ratios and 95% confidence intervals for renal cell carcinoma risk in the EPIC study, among women who reported weight at age 20 (n = 137,826)
**Table S11:** Hazard ratios and 95% confidence intervals for renal cell carcinoma risk in the EPIC study, adjusted for hypertension status (n = 251,963)

## Data Availability

For information on how to submit an application for gaining access to EPIC data and/or biospecimens, please follow the instructions at http://epic.iarc.fr/access/index.

## References

[1] Cancer Today [Internet] . [cited 2022 Feb 1]. Available from: https://gco.iarc.fr/today/online‐analysis‐multi‐bars?v=2020&mode=cancer&mode_population=countries&population=900&populations=908&key=asr&sex=2&cancer=39&type=0&statistic=5&prevalence=0&population_group=0&ages_group%5B%5D=0&ages_group%5B%5D=17&nb_items=10&group_cancer=1&include_nmsc=1&include_nmsc_other=1&type_multiple=%257B%2522inc%2522%253Atrue%252C%2522mort%2522%253Afalse%252C%2522prev%2522%253Afalse%257D&orientation=horizontal&type_sort=0&type_nb_items=%257B%2522top%2522%253Atrue%252C%2522bottom%2522%253Afalse%257D#collapse‐group‐0‐4

[2] Chow WH , Dong LM , Devesa SS . Epidemiology and risk factors for kidney cancer. Nat Rev Urol. 2010;7(5):245‐257. 20448658 10.1038/nrurol.2010.46 PMC3012455

[3] Lipworth L , Morgans AK , Edwards TL , et al. Renal cell cancer histological subtype distribution differs by race and sex. BJU Int. 2016;117(2):260‐265. 25307281 10.1111/bju.12950

[4] Liang J , Shang Y . Estrogen and Cancer. Annu Rev Physiol. 2013;75:225‐240. 23043248 10.1146/annurev-physiol-030212-183708

[5] Troisi R , Bjørge T , Gissler M , et al. The role of pregnancy, perinatal factors and hormones in maternal cancer risk: a review of the evidence. J Intern Med. 2018;283(5):430‐445. 29476569 10.1111/joim.12747 PMC6688839

[6] Cheung KL , Lafayette RA . Renal physiology of pregnancy. Adv Chronic Kidney Dis. 2013;20(3):209‐214. 23928384 10.1053/j.ackd.2013.01.012 PMC4089195

[7] Calle EE , Kaaks R . Overweight, obesity and cancer: epidemiological evidence and proposed mechanisms. Nat Rev Cancer. 2004;4(8):579‐591. 15286738 10.1038/nrc1408

[8] Riboli E , Kaaks R . The EPIC project: rationale and study design. European prospective investigation into cancer and nutrition. Int J Epidemiol. 1997;26(90001):S6‐S14. 9126529 10.1093/ije/26.suppl_1.s6

[9] Riboli E , Hunt KJ , Slimani N , et al. European prospective investigation into cancer and nutrition (EPIC): study populations and data collection. Public Health Nutr. 2002;5:1113‐1124. 12639222 10.1079/PHN2002394

[10] Lahmann PH , Hoffmann K , Allen N , et al. Body size and breast cancer risk: findings from the European prospective investigation into cancer and nutrition (EPIC). Int J Cancer. 2004;111(5):762‐771. 15252848 10.1002/ijc.20315

[11] Kabat GC , Navarro Silvera SA , Miller AB , Rohan TE . A cohort study of reproductive and hormonal factors and renal cell cancer risk in women. Br J Cancer. 2007;96(5):845‐849. 17311018 10.1038/sj.bjc.6603629 PMC2360073

[12] Molokwu JC , Prizment AE , Folsom AR . Reproductive characteristics and risk of kidney cancer: Iowa Women's health study. Maturitas. 2007;58(2):156‐163. 17822863 10.1016/j.maturitas.2007.07.003

[13] Lee JE , Hankinson SE , Cho E . Reproductive factors and risk of renal cell CancerThe Nurses' health study. Am J Epidemiol. 2009;169(10):1243‐1250. 19329527 10.1093/aje/kwp030 PMC2727207

[14] Schouten LJ , van de Pol J , Kviatkovsky MJ , van den Brandt PA . Reproductive and external hormonal factors and the risk of renal cell cancer in The Netherlands cohort study. Cancer Epidemiol. 2022;6:79. 10.1016/j.canep.2022.102171 35533551

[15] Karami S , Daugherty SE , Schonfeld SJ , et al. Reproductive factors and kidney cancer risk in 2 US cohort studies, 1993–2010. Am J Epidemiol. 2013;177(12):1368‐1377. 23624999 10.1093/aje/kws406 PMC3676151

[16] Guan HB , Wu QJ , Gong TT . Parity and kidney cancer risk: evidence from epidemiologic studies. Cancer Epidemiology and Prevention Biomarkers. 2013;22(12):2345‐2353. 10.1158/1055-9965.EPI-13-0759-T 24108791

[17] Lambe M , Lindblad P , Wuu J , Remler R , Hsieh CC . Pregnancy and risk of renal cell cancer: a population‐based study in Sweden. British Journal of Cancer. 2002;86:1425‐1429. 11986775 10.1038/sj.bjc.6600263 PMC2375385

[18] Chiu HF , Kuo CC , Kuo HW , Lee IM , Yang CY . Parity, age at first birth and risk of death from kidney cancer: a population‐based cohort study in Taiwan. Eur J Public Health. 2014;24(2):249‐252. 23748595 10.1093/eurpub/ckt057

[19] Karami S , Daugherty SE , Purdue MP . Hysterectomy and kidney cancer risk: a meta‐analysis. Int J Cancer. 2014;134(2):405‐410. 23818138 10.1002/ijc.28352 PMC3834077

[20] Luo J , Rohan TE , Neuhouser ML , et al. Hysterectomy, oophorectomy, and risk of renal cell carcinoma. Cancer Epidemiol Biomarkers Prev. 2020;30(3):499‐506. 33335021 10.1158/1055-9965.EPI-20-1373

[21] Wilson LF , Tuesley KM , Webb PM , Dixon‐Suen SC , Stewart LM , Jordan SJ . Hysterectomy and risk of breast, colorectal, thyroid, and kidney cancer – an Australian data linkage study. Cancer Epidemiol Biomarkers Prev. 2021;30(5):904‐911. 33619026 10.1158/1055-9965.EPI-20-1670

[22] Liu H , Wang XC , Hu GH , Huang TB , Xu YF . Oral contraceptive use and kidney cancer risk among women: evidence from a meta‐analysis. Int J Clin Exp Med. 2014;7(11):3954‐3963. 25550903 PMC4276161

[23] Zhang X , Du Y , Tan X , et al. The relationship between hormone replacement therapy and risk of kidney cancer in women: a meta‐analysis. Cancer Control. 2020;27(2): 1073274820930194. 10.1177/1073274820930194 PMC7366413 32668959

[24] Phipps AI , Buist DSM . Validation of self‐reported history of hysterectomy and oophorectomy among women in an integrated group practice setting. Menopause. 2009;16:576‐581. 19169161 10.1097/gme.0b013e31818ffe28 PMC2695678

[25] Christin‐Maitre S . History of oral contraceptive drugs and their use worldwide. Best Pract Res Clin Endocrinol Metab. 2013;27(1):3‐12. 23384741 10.1016/j.beem.2012.11.004

[26] Sutton CJG . The history of hysterectomy. The History of Hysterectomy. A Comprehensive Surgical Approach; 2018:3‐28.

[27] Ashraf MS , Vongpatanasin W . Estrogen and Hypertension. Curr Hypertens Rep. 2006;8:368‐376. 16965722 10.1007/s11906-006-0080-1

