# Supplementary material for: Reproductive and hormonal factors and risk of renal cell carcinoma among women in the European Prospective Investigation into Cancer and Nutrition
Source: Cancer Med. 2023 Jun 3;12(14):15588–600. doi: 10.1002/cam4.6207 (PMC10417104; doi:10.1002/cam4.6207)
Supplement: Supplementary file 1 — Table S1: Histological subtypes of renal cell carcinoma cases in the EPIC study Table S2: Hazard ratios and 95% confidence intervals for clear cell renal cell carcinoma risk by age at menarche, menopause status, and age at menopause in the EPIC study Table S3: Hazard ratios and 95% confidence intervals for clear cell renal cell carcinoma risk by reproductive factors in the EPIC study Table S4: Hazard ratios and 95% confidence intervals for clear cell renal cell carcinoma risk by hysterectomy and ovariectomy status in the EPIC study Table S5: Hazard ratios and 95% confidence intervals for clear cell renal cell carcinoma risk by exogenous hormone use in the EPIC study Table S6: Hazard ratios and 95% confidence intervals for renal cell carcinoma risk by age at menarche, menopause status, and age at menopause in the EPIC study, excluding the first three years of follow‐up Table S7: Hazard ratios and 95% confidence intervals for renal cell carcinoma risk by reproductive factors in the EPIC study, excluding the first three years of follow‐up Table S8: Hazard ratios and 95% confidence intervals for renal cell carcinoma risk by hysterectomy and ovariectomy status in the EPIC study, excluding the first three years of follow‐up Table S9: Hazard ratios and 95% confidence intervals for renal cell carcinoma risk by exogenous hormone use in the EPIC study, excluding the first three years of follow‐up Table S10: Hazard ratios and 95% confidence intervals for renal cell carcinoma risk in the EPIC study, among women who reported weight at age 20 (n = 137,826) Table S11: Hazard ratios and 95% confidence intervals for renal cell carcinoma risk in the EPIC study, adjusted for hypertension status (n = 251,963) [file CAM4-12-15588-s001.docx]

# Supplementary Materials

Supplementary Table 1: Histological subtypes of renal cell carcinoma cases in the EPIC study

| RCC subtype | Number of cases |
| --- | --- |
| clear cell | 156 |
| papillary | 13 |
| chromophobe | 10 |
| other | 15 |
| NOS | 244 |
| Abbreviations: Not otherwise specified (NOS) | |

Supplementary Table 2: Hazard ratios and 95% confidence intervals for clear cell renal cell carcinoma risk by age at menarche, menopause status, and age at menopause in the EPIC study

| Exposure | Category | Number of cases | Adjusted for BMI, smoking status, and education HR (95% CI)^1^ | Additionally adjusted for age at menarche HR (95% CI)^2^ | Restricted to women without hysterectomy or bilateral ovariectomy Number of cases HR (95% CI)^2^ | |
| --- | --- | --- | --- | --- | --- | --- |
| Age at menarche (years) | < 12 | 19 | Reference |  |  |  |
|  | 12 | 29 | 1.06 (0.59, 1.89) |  |  |  |
|  | 13 | 32 | 0.96 (0.54, 1.71) |  |  |  |
|  | 14 | 43 | 1.43 (0.82, 2.50) |  |  |  |
|  | 15+ | 33 | 1.37 (0.76, 2.47) |  |  |  |
| Menopause status | Premenopausal | 35 | Reference | Reference | 31 | Reference |
|  | Perimenopausal | 32 | 0.76 (0.43, 1.35) | 0.76 (0.43, 1.34) | 25 | 0.99 (0.52, 1.87) |
|  | Postmenopausal | 89 | 0.53 (0.30, 0.94) | 0.52 (0.29, 0.93) | 53 | 0.54 (0.28, 1.06) |
| Among postmenopausal women | |  |  |  |  |  |
| Age at menopause (years) | < 46 | 18 | Reference | Reference | 7 | Reference |
|  | 46 - 48 | 14 | 0.90 (0.45, 1.82) | 0.89 (0.44, 1.79) | 9 | 1.01 (0.38, 2.73) |
|  | 49 - 51 | 19 | 0.71 (0.37, 1.37) | 0.70 (0.37, 1.34) | 14 | 0.82 (0.33, 2.05) |
|  | 52 - 54 | 19 | 0.96 (0.50, 1.84) | 0.94 (0.49, 1.79) | 11 | 0.79 (0.30, 2.06) |
|  | 55+ | 5 | 0.66 (0.24, 1.81) | 0.64 (0.24, 1.74) | 4 | 0.78 (0.22, 2.68) |
| ^1^Stratified by country and adjusted for BMI, smoking status, and education | | | | | | |
| ^2^Stratified by country and adjusted for BMI, smoking status, education, and age at menarche | | | | | | |
|  | | | | | | |

Supplementary Table 3: Hazard ratios and 95% confidence intervals for clear cell renal cell carcinoma risk by reproductive factors in the EPIC study

| Exposure | Category | Number of cases | Adjusted for BMI, smoking status, and education HR (95% CI)^1^ | Additionally adjusted for age at menarche and menopause status HR (95% CI)^2^ | Mutually adjusted HR (95% CI)^3^ |
| --- | --- | --- | --- | --- | --- |
| Full-term pregnancy | No | 10 | Reference | Reference |  |
|  | Yes | 145 | 1.78 (0.93, 3.40) | 1.76 (0.92, 3.36) |  |
| Among parous women only |  |  |  |  |  |
| Number of full-term pregnancies | 1 | 15 | Reference | Reference | Reference |
|  | 2 | 70 | 1.76 (1.01, 3.09) | 1.75 (1.00, 3.07) | 1.66 (0.93, 2.98) |
|  | 3 | 29 | 1.38 (0.73, 2.60) | 1.38 (0.73, 2.60) | 1.39 (0.71, 2.72) |
|  | 4+ | 21 | 1.81 (0.91, 3.61) | 1.83 (0.92, 3.65) | 1.85 (0.88, 3.92) |
| Age at first full-term pregnancy (years) | < 20 | 19 | Reference | Reference | Reference |
|  | 20 - 24 | 57 | 0.60 (0.35, 1.02) | 0.59 (0.35, 1.00) | 0.61 (0.36, 1.06) |
|  | 25 - 29 | 46 | 0.59 (0.33, 1.04) | 0.58 (0.33, 1.03) | 0.62 (0.34, 1.12) |
|  | 30+ | 13 | 0.45 (0.22, 0.95) | 0.44 (0.21, 0.93) | 0.53 (0.25, 1.17) |
| Total duration of breastfeeding (months) | 0 | 14 | Reference | Reference | Reference |
|  | < 3 | 27 | 1.34 (0.70, 2.56) | 1.33 (0.70, 2.56) | 1.28 (0.67, 2.46) |
|  | 3 - 8 | 45 | 1.36 (0.74, 2.50) | 1.36 (0.74, 2.50) | 1.26 (0.68, 2.31) |
|  | 9+ | 46 | 1.13 (0.61, 2.09) | 1.12 (0.60, 2.07) | 0.97 (0.51, 1.83) |
| ^1^Stratified by country and adjusted for BMI, smoking status, and education | | | | | |
| ^2^Stratified by country and adjusted for BMI, smoking status, education, age at menarche, and menopause status | | | | | |
| ^3^Stratified by country and adjusted for BMI, smoking status, education, age at menarche, menopause status, and mutually adjusted for the other pregnancy-related exposures | | | | | |

Supplementary Table 4: Hazard ratios and 95% confidence intervals for clear cell renal cell carcinoma risk by hysterectomy and ovariectomy status in the EPIC study

| Exposure | Category | Number of cases | Adjusted for BMI, smoking status, and education HR (95% CI)^1^ | Additionally adjusted for age at menarche HR (95% CI)^2^ | Mutually adjusted HR (95% CI)^3^ |
| --- | --- | --- | --- | --- | --- |
| Hysterectomy | No | 89 | Reference | Reference | Reference |
|  | Yes | 17 | 1.01 (0.60, 1.72) | 1.02 (0.60, 1.73) | 1.15 (0.61, 2.15) |
| Ovariectomy | No | 97 | Reference | Reference | Reference |
|  | Bilateral | 5 | 0.88 (0.36, 2.18) | 0.88 (0.36, 2.18) | 0.79 (0.28, 2.24) |
|  | Unilateral | 4 | 0.71 (0.26, 1.95) | 0.72 (0.26, 1.95) | 0.67 (0.24, 1.91) |
| ^1^Stratified by country and adjusted for BMI, smoking status, and education | | | | | |
| ^2^Stratified by country and adjusted for BMI, smoking status, education, and age at menarche | | | | | |
| ^3^Stratified by country and adjusted for BMI, smoking status, education, age at menarche, and mutually adjusted for ovariectomy or hysterectomy | | | | | |

Supplementary Table 5: Hazard ratios and 95% confidence intervals for clear cell renal cell carcinoma risk by exogenous hormone use in the EPIC study

| Exposure | Category | Number of cases | Adjusted for BMI, smoking status, and education HR (95% CI)^1^ | Additionally adjusted for age at menarche and menopause status HR (95% CI)^2^ | Mutually adjusted HR (95% CI)^3^ |
| --- | --- | --- | --- | --- | --- |
| Ever use of OC pill | No | 69 | Reference | Reference | Reference |
|  | Yes | 80 | 1.15 (0.81, 1.63) | 1.11 (0.78, 1.58) | 1.12 (0.78, 1.60) |
| HRT | Never | 114 | Reference | Reference | Reference |
|  | Past | 7 | 0.49 (0.23, 1.06) | 0.53 (0.24, 1.14) | 0.52 (0.24, 1.13) |
|  | Current | 28 | 0.89 (0.57, 1.39) | 1.04 (0.65, 1.65) | 1.02 (0.64, 1.64) |
| Among OC ever users |  |  |  |  |  |
| Duration of OC pill use (years) | < 2 | 23 | Reference | Reference |  |
|  | 2 - 5 | 24 | 0.81 (0.45, 1.44) | 0.80 (0.45, 1.42) |  |
|  | 6 - 10 | 19 | 0.90 (0.48, 1.68) | 0.88 (0.47, 1.65) |  |
|  | 11+ | 12 | 0.53 (0.25, 1.11) | 0.53 (0.25, 1.11) |  |
| Among HRT past or current users |  |  |  |  |  |
| Duration of HRT use (years) | < 2 | 13 | Reference | Reference |  |
|  | 2 - 5 | 10 | 0.62 (0.26, 1.45) | 0.73 (0.30, 1.73) |  |
|  | 6+ | 11 | 1.14 (0.48, 2.75) | 1.41 (0.57, 3.46) |  |
| ^1^Stratified by country and adjusted for BMI, smoking status, and education | | | | | |
| ^2^Stratified by country and adjusted for BMI, smoking status, education, age at menarche, and menopause status | | | | | |
| ^3^Stratified by country and adjusted for BMI, smoking status, education, age at menarche, menopause status, and mutually adjusted for the HRT or OC use | | | | | |

Supplementary Table 6: Hazard ratios and 95% confidence intervals for renal cell carcinoma risk by age at menarche, menopause status, and age at menopause in the EPIC study, excluding the first three years of follow-up

| Exposure | Category | Number of cases | Adjusted for BMI, smoking status, and education HR (95% CI)^1^ | Additionally adjusted for age at menarche HR (95% CI)^2^ | Restricted to women without hysterectomy or bilateral ovariectomy Number of cases HR (95% CI)^2^ | |
| --- | --- | --- | --- | --- | --- | --- |
| Age at menarche (years) | < 12 | 56 | Reference |  |  |  |
|  | 12 | 64 | 0.80 (0.56, 1.15) |  |  |  |
|  | 13 | 87 | 0.85 (0.60, 1.19) |  |  |  |
|  | 14 | 84 | 0.86 (0.61, 1.22) |  |  |  |
|  | 15+ | 78 | 0.94 (0.65, 1.34) |  |  |  |
| Menopause status | Premenopausal | 56 | Reference | Reference | 51 | Reference |
|  | Perimenopausal | 68 | 1.02 (0.67, 1.57) | 1.02 (0.67, 1.57) | 47 | 1.25 (0.78, 2.01) |
|  | Postmenopausal | 245 | 0.85 (0.56, 1.31) | 0.85 (0.56, 1.31) | 143 | 0.85 (0.53, 1.39) |
| Among postmenopausal women | |  |  |  |  |  |
| Age at menopause (years) | < 46 | 56 | Reference | Reference | 19 | Reference |
|  | 46 - 48 | 37 | 0.79 (0.52, 1.20) | 0.79 (0.52, 1.20) | 22 | 0.93 (0.50, 1.73) |
|  | 49 - 51 | 49 | 0.63 (0.43, 0.92) | 0.62 (0.42, 0.92) | 32 | 0.71 (0.40, 1.26) |
|  | 52 - 54 | 49 | 0.85 (0.57, 1.25) | 0.84 (0.57, 1.24) | 33 | 0.93 (0.52, 1.64) |
|  | 55+ | 15 | 0.67 (0.37, 1.18) | 0.66 (0.37, 1.17) | 12 | 0.88 (0.42, 1.82) |
| ^1^Stratified by country and adjusted for BMI, smoking status, and education | | | | | | |
| ^2^Stratified by country and adjusted for BMI, smoking status, education, and age at menarche | | | | | | |
|  | | | | | | |

Supplementary Table 7: Hazard ratios and 95% confidence intervals for renal cell carcinoma risk by reproductive factors in the EPIC study, excluding the first three years of follow-up

| Exposure | Category | Number of cases | Adjusted for BMI, smoking status, and education HR (95% CI)^1^ | Additionally adjusted for age at menarche and menopause status HR (95% CI)^2^ | Mutually adjusted HR (95% CI)^3^ |
| --- | --- | --- | --- | --- | --- |
| Full-term pregnancy | No | 25 | Reference | Reference |  |
|  | Yes | 341 | 1.77 (1.18, 2.67) | 1.77 (1.17, 2.66) |  |
| Among parous women only |  |  |  |  |  |
| Number of full-term pregnancies | 1 | 56 | Reference | Reference | Reference |
|  | 2 | 147 | 1.01 (0.74, 1.38) | 1.01 (0.74, 1.38) | 0.98 (0.71, 1.37) |
|  | 3 | 71 | 0.94 (0.65, 1.34) | 0.94 (0.65, 1.34) | 0.92 (0.63, 1.36) |
|  | 4+ | 44 | 1.08 (0.71, 1.63) | 1.08 (0.72, 1.64) | 1.10 (0.70, 1.74) |
| Age at first full-term pregnancy (years) | < 20 | 42 | Reference | Reference | Reference |
|  | 20 - 24 | 117 | 0.59 (0.41, 0.85) | 0.59 (0.41, 0.85) | 0.60 (0.41, 0.86) |
|  | 25 - 29 | 128 | 0.80 (0.55, 1.16) | 0.80 (0.55, 1.16) | 0.80 (0.54, 1.17) |
|  | 30+ | 31 | 0.53 (0.33, 0.87) | 0.54 (0.33, 0.87) | 0.52 (0.31, 0.88) |
| Total duration of breastfeeding (months) | 0 | 42 | Reference | Reference | Reference |
|  | < 3 | 62 | 0.92 (0.62, 1.36) | 0.92 (0.62, 1.36) | 0.91 (0.61, 1.35) |
|  | 3 - 8 | 102 | 0.94 (0.65, 1.35) | 0.94 (0.65, 1.35) | 0.92 (0.64, 1.33) |
|  | 9+ | 105 | 0.82 (0.57, 1.19) | 0.82 (0.57, 1.19) | 0.80 (0.54, 1.18) |
| ^1^Stratified by country and adjusted for BMI, smoking status, and education | | | | | |
| ^2^Stratified by country and adjusted for BMI, smoking status, education, age at menarche, and menopause status | | | | | |
| ^3^Stratified by country and adjusted for BMI, smoking status, education, age at menarche, menopause status, and mutually adjusted for the other pregnancy-related exposures | | | | | |

Supplementary Table 8: Hazard ratios and 95% confidence intervals for renal cell carcinoma risk by hysterectomy and ovariectomy status in the EPIC study, excluding the first three years of follow-up

| Exposure | Category | Number of cases | Adjusted for BMI, smoking status, and education HR (95% CI)^1^ | Additionally adjusted for age at menarche HR (95% CI)^2^ | Mutually adjusted HR (95% CI)^3^ |
| --- | --- | --- | --- | --- | --- |
| Hysterectomy | No | 212 | Reference | Reference | Reference |
|  | Yes | 62 | 1.51 (1.13, 2.02) | 1.51 (1.13, 2.01) | 1.36 (0.95, 1.95) |
| Ovariectomy | No | 234 | Reference | Reference | Reference |
|  | Bilateral | 24 | 1.76 (1.15, 2.68) | 1.75 (1.15, 2.68) | 1.36 (0.82, 2.28) |
|  | Unilateral | 16 | 1.16 (0.70, 1.92) | 1.15 (0.69, 1.92) | 1.00 (0.59, 1.72) |
| ^1^Stratified by country and adjusted for BMI, smoking status, and education | | | | | |
| ^2^Stratified by country and adjusted for BMI, smoking status, education, and age at menarche | | | | | |
| ^3^Stratified by country and adjusted for BMI, smoking status, education, age at menarche, and mutually adjusted for ovariectomy or hysterectomy | | | | | |

Supplementary Table 9: Hazard ratios and 95% confidence intervals for renal cell carcinoma risk by exogenous hormone use in the EPIC study, excluding the first three years of follow-up

| Exposure | Category | Number of cases | Adjusted for BMI, smoking status, and education HR (95% CI)^1^ | Additionally adjusted for age at menarche and menopause status HR (95% CI)^2^ | Mutually adjusted HR (95% CI)^3^ |
| --- | --- | --- | --- | --- | --- |
| Ever use of OC pill | No | 176 | Reference | Reference | Reference |
|  | Yes | 168 | 0.97 (0.77, 1.22) | 0.96 (0.76, 1.21) | 0.97 (0.76, 1.22) |
| HRT | Never | 241 | Reference | Reference | Reference |
|  | Past | 39 | 1.05 (0.74, 1.49) | 1.07 (0.75, 1.51) | 1.07 (0.76, 1.52) |
|  | Current | 64 | 0.87 (0.65, 1.17) | 0.90 (0.66, 1.22) | 0.90 (0.67, 1.23) |
| Among OC ever users |  |  |  |  |  |
| Duration of OC pill use (years) | < 2 | 41 | Reference | Reference |  |
|  | 2 - 5 | 41 | 0.75 (0.48, 1.16) | 0.75 (0.48, 1.16) |  |
|  | 6 - 10 | 49 | 1.16 (0.76, 1.79) | 1.16 (0.76, 1.79) |  |
|  | 11+ | 31 | 0.66 (0.40, 1.08) | 0.66 (0.40, 1.08) |  |
| Among HRT past or current users |  |  |  |  |  |
| Duration of HRT use (years) | < 2 | 38 | Reference | Reference |  |
|  | 2 - 5 | 27 | 0.62 (0.37, 1.04) | 0.68 (0.40, 1.13) |  |
|  | 6+ | 31 | 1.00 (0.59, 1.67) | 1.09 (0.64, 1.85) |  |
| ^1^Stratified by country and adjusted for BMI, smoking status, and education | | | | | |
| ^2^Stratified by country and adjusted for BMI, smoking status, education, age at menarche, and menopause status | | | | | |
| ^3^Stratified by country and adjusted for BMI, smoking status, education, age at menarche, menopause status, and mutually adjusted for HRT or OC use | | | | | |

Supplementary Table 10: Hazard ratios and 95% confidence intervals for renal cell carcinoma risk in the EPIC study, among women who reported weight at age 20 (n = 137,826)

| Population | Exposure | Category | Number of cases | Adjusted for BMI at baseline HR (95% CI)^1^ | Adjusted for BMI at age 20 HR (95% CI)^2^ |
| --- | --- | --- | --- | --- | --- |
|  | Age at menarche (years) | < 12 | 30 | Reference | Reference |
|  |  | 12 | 41 | 0.97 (0.60, 1.56) | 0.94 (0.58, 1.50) |
|  |  | 13 | 54 | 0.94 (0.59, 1.47) | 0.89 (0.56, 1.39) |
|  |  | 14 | 47 | 0.82 (0.51, 1.32) | 0.77 (0.48, 1.24) |
|  |  | 15+ | 52 | 1.05 (0.65, 1.67) | 0.97 (0.61, 1.55) |
|  | Menopause status | Premenopausal | 30 | Reference | Reference |
|  |  | Perimenopausal | 37 | 0.75 (0.43, 1.29) | 0.77 (0.44, 1.33) |
|  |  | Postmenopausal | 157 | 0.78 (0.46, 1.32) | 0.80 (0.47, 1.35) |
| Among postmenopausal women | Age at menopause (years) | < 46 | 30 | Reference | Reference |
|  |  | 46 - 48 | 20 | 0.77 (0.44, 1.36) | 0.76 (0.43, 1.33) |
|  |  | 49 - 51 | 32 | 0.75 (0.45, 1.24) | 0.74 (0.45, 1.22) |
|  |  | 52 - 54 | 37 | 1.24 (0.76, 2.03) | 1.24 (0.76, 2.01) |
|  |  | 55+ | 11 | 0.90 (0.45, 1.81) | 0.91 (0.45, 1.83) |
|  | Full-term pregnancy | No | 17 | Reference | Reference |
|  |  | Yes | 205 | 1.44 (0.88, 2.38) | 1.48 (0.90, 2.44) |
| Among parous women | Number of full-term pregnancies | 1 | 36 | Reference | Reference |
|  |  | 2 | 97 | 1.01 (0.69, 1.48) | 1.01 (0.69, 1.49) |
|  |  | 3 | 47 | 0.93 (0.60, 1.44) | 0.94 (0.61, 1.47) |
|  |  | 4+ | 24 | 1.09 (0.64, 1.84) | 1.16 (0.69, 1.95) |
|  | Age at first full-term pregnancy (years) | < 20 | 35 | Reference | Reference |
|  |  | 20 - 24 | 86 | 0.66 (0.44, 0.99) | 0.64 (0.43, 0.95) |
|  |  | 25 - 29 | 68 | 0.78 (0.51, 1.21) | 0.74 (0.48, 1.14) |
|  |  | 30+ | 15 | 0.47 (0.25, 0.88) | 0.44 (0.23, 0.82) |
|  | Total duration of breastfeeding (months) | 0 | 19 | Reference | Reference |
|  |  | < 3 | 38 | 1.10 (0.63, 1.92) | 1.08 (0.62, 1.89) |
|  |  | 3 - 8 | 72 | 1.09 (0.65, 1.81) | 1.06 (0.64, 1.78) |
|  |  | 9+ | 70 | 0.91 (0.54, 1.53) | 0.90 (0.54, 1.51) |
|  | Hysterectomy | No | 115 | Reference | Reference |
|  |  | Yes | 33 | 1.53 (1.03, 2.26) | 1.56 (1.05, 2.30) |
|  | Ovariectomy | No | 130 | Reference | Reference |
|  |  | Bilateral | 12 | 1.52 (0.84, 2.76) | 1.57 (0.87, 2.85) |
|  |  | Unilateral | 6 | 0.75 (0.33, 1.70) | 0.76 (0.33, 1.71) |
|  | Ever use of OC pill | No | 109 | Reference | Reference |
|  |  | Yes | 98 | 0.89 (0.66, 1.20) | 0.88 (0.66, 1.18) |
|  | HRT | Never | 138 | Reference | Reference |
|  |  | Past | 24 | 0.94 (0.61, 1.47) | 0.96 (0.61, 1.49) |
|  |  | Current | 45 | 0.79 (0.56, 1.12) | 0.77 (0.54, 1.09) |
| Among OC ever users | Duration of OC pill use (years) | < 2 | 19 | Reference | Reference |
|  |  | 2 - 5 | 33 | 1.23 (0.69, 2.17) | 1.23 (0.70, 2.18) |
|  |  | 6 - 10 | 26 | 1.39 (0.76, 2.56) | 1.39 (0.76, 2.55) |
|  |  | 11+ | 17 | 0.87 (0.44, 1.74) | 0.87 (0.44, 1.74) |
| Among HRT past or current users | Duration of HRT use (years) | < 2 | 19 | Reference | Reference |
|  |  | 2 - 5 | 17 | 0.66 (0.34, 1.30) | 0.66 (0.34, 1.28) |
|  |  | 6+ | 26 | 1.34 (0.72, 2.50) | 1.33 (0.71, 2.48) |
| ^1^Stratified by country and adjusted for BMI at baseline, smoking status, and education | | | | | |
| ^2^Stratified by country and adjusted for BMI at age 20 years, smoking status, and education | | | | | |

Supplementary Table 11: Hazard ratios and 95% confidence intervals for renal cell carcinoma risk in the EPIC study, adjusted for hypertension status (n = 251,963)

| Population | Predictor | Category | Number of cases | Adjusted for hypertension HR (95% CI) |
| --- | --- | --- | --- | --- |
|  | Age at menarche (years) | < 12 | 53 | Reference |
|  |  | 12 | 65 | 0.89 (0.62, 1.29) |
|  |  | 13 | 75 | 0.82 (0.58, 1.18) |
|  |  | 14 | 83 | 0.98 (0.69, 1.40) |
|  |  | 15+ | 65 | 0.92 (0.63, 1.34) |
|  | Menopause status | Premenopausal | 57 | Reference |
|  |  | Perimenopausal | 60 | 1.05 (0.68, 1.61) |
|  |  | Postmenopausal | 224 | 0.83 (0.55, 1.27) |
| Among postmenopausal women | Age at menopause (years) | < 46 | 50 | Reference |
|  |  | 46 - 48 | 35 | 0.85 (0.55, 1.31) |
|  |  | 49 - 51 | 45 | 0.65 (0.43, 0.98) |
|  |  | 52 - 54 | 44 | 0.89 (0.59, 1.33) |
|  |  | 55+ | 15 | 0.74 (0.41, 1.32) |
|  | Full term pregnancy | No | 21 | Reference |
|  |  | Yes | 317 | 1.98 (1.27, 3.08) |
| Among parous women | Number of full term pregnancies | 1 | 53 | Reference |
|  |  | 2 | 139 | 1.06 (0.77, 1.46) |
|  |  | 3 | 61 | 0.91 (0.62, 1.32) |
|  |  | 4+ | 37 | 1.03 (0.66, 1.60) |
|  | Age at first full term pregnancy (years) | < 20 | 45 | Reference |
|  |  | 20 - 24 | 105 | 0.49 (0.34, 0.70) |
|  |  | 25 - 29 | 109 | 0.64 (0.44, 0.92) |
|  |  | 30+ | 31 | 0.49 (0.31, 0.80) |
|  | Total duration of breastfeeding (months) | 0 | 35 | Reference |
|  |  | < 3 | 58 | 1.07 (0.70, 1.63) |
|  |  | 3 - 8 | 96 | 1.08 (0.73, 1.60) |
|  |  | 9+ | 97 | 0.93 (0.63, 1.39) |
|  | Hysterectomy | No | 211 | Reference |
|  |  | Yes | 59 | 1.48 (1.10, 1.99) |
|  | Ovariectomy | No | 231 | Reference |
|  |  | Bilateral | 25 | 1.73 (1.14, 2.63) |
|  |  | Unilateral | 14 | 1.02 (0.59, 1.75) |
|  | Ever use of OC pill | No | 173 | Reference |
|  |  | Yes | 151 | 0.91 (0.72, 1.16) |
|  | HRT | Never | 223 | Reference |
|  |  | Past | 35 | 1.02 (0.70, 1.46) |
|  |  | Current | 66 | 1.06 (0.78, 1.42) |
| Among OC ever users | Duration of OC pill use (years) | < 2 | 34 | Reference |
|  |  | 2 - 5 | 40 | 0.93 (0.59, 1.48) |
|  |  | 6 - 10 | 42 | 1.35 (0.85, 2.16) |
|  |  | 11+ | 31 | 0.88 (0.52, 1.49) |
| Among HRT past or current users | Duration of HRT use (years) | < 2 | 37 | Reference |
|  |  | 2 - 5 | 28 | 0.68 (0.41, 1.14) |
|  |  | 6+ | 29 | 0.87 (0.51, 1.47) |
| Stratified by country and adjusted for hypertension, smoking status, and education | | | | |
